# Measuring Nutrition and Food Literacy in Adults: A Systematic Review and Appraisal of Existing Measurement Tools

**DOI:** 10.3928/24748307-20180625-01

**Published:** 2018-08-15

**Authors:** Eva Y. N. Yuen, Maria Thomson, Heather Gardiner

## Abstract

**Background:**

Nutrition literacy (NL) and food literacy (FL) have emerged as key components in the promotion and maintenance of healthy dietary practices. However, a critical appraisal of existing tools is required to advance the operationalization and measurement of these constructs using instruments that demonstrate sound validity and reliability.

**Methods:**

Electronic databases were searched in January and July 2016, January 2017, and March 2018 for publications detailing the development and/or testing of NL or FL instruments. Instruments' psychometric properties were assessed using a structured methodological framework. We identified 2,563 new titles and abstracts, and short-listed 524 for full review. The extent to which key domains of NL were included in each measure was examined.

**Key Results:**

Thirteen instruments assessing NL underwent full evaluation; seven from the United States, and one each from Australia, Norway, Switzerland, Italy, Hong Kong, and Japan. Measures targeted general Spanish-, Italian-, or Cantonese-speaking adults; primary care patients, parent, and populations with breast cancer. Instruments ranged from 6 to 64 items, and they predominantly assessed functional NL rather than broader domains of NL. Substantial variation in methodological rigor was observed across measures.

**Discussion:**

Multidimensional and psychometrically sound measures that capture broader domains of NL and assess FL are needed.

**Plain Language Summary:**

This review systemically compiles, and critically appraises 13 existing measures that assess nutrition literacy and food literacy in an adult population. Substantial variation in methodological rigor was found across the measures, and most tools assessed nutrition literacy rather than food literacy. Findings from this current review may be useful to guide development of future measures that comprehensively capture nutrition literacy and food literacy. **[*HLRP: Health Literacy Research and Practice***. **2018;2(3):e134–e160.]**

Adequate nutrition knowledge, optimal dietary behaviors, and the maintenance of a healthy weight are now recognized as key modifiable factors in health promotion and chronic disease prevention (Bauer, Briss, Goodman, & Bowman, 2014; Mozaffarian, 2016). Yet, despite the 2015 to 2020 Dietary Guidelines for Americans (US Department of Health and Human Services, 2017) recommending that adults consume at least 1.5 to 2 cups of fruits and 2 to 3 cups of vegetables daily, it is estimated that only 13.1% of adults in the United States met fruit recommendations and only 8.9% met vegetable recommendations (Moore & Thompson, 2015). Moreover, inadequate nutrition and dietary practices are major contributors to obesity, diabetes, cancer, and cardiovascular disease (Arnold et al., 2015; Garg, Maurer, & Reed, 2014; Mozaffarian, 2016).

Health literacy (HL) is broadly defined as a person's knowledge, motivation, and competencies enabling the identification, appraisal, and application of health information to make health decisions (Sørensen et al., 2012). Inadequate HL has been associated with poorer self-management of chronic health conditions, including cardiovascular disease (Gazmararian, Kripalani, & Miller, 2006; Kripalani, Gatti, & Jacobson, 2010), asthma (Apter et al., 2013; Federman et al., 2014), diabetes (van der Heide et al., 2014), and increased morbidity and mortality (Baker et al., 2007; Moser et al., 2015). Two specific types of HL, nutrition literacy (NL) and food literacy (FL), have emerged as key components in the promotion and maintenance of healthy dietary practices (Cullen, Hatch, Martin, Higgins, & Sheppard, 2015; Krause, Sommerhalder, Beer-Borst, & Abel, 2018b; Velardo, 2015). Whereas NL has been defined as the capacity to obtain, process, and understand nutrition information and skills needed to make appropriate nutrition decisions (Silk et al., 2008), FL is described as going beyond nutrition knowledge to include the application of nutritional information to make food choices and to critically appraise personal and societal dietary behaviors (Krause, Sommerhalder, & Beer-Borst, 2016). Specifically, definitions of FL have included broader components including food preparation and food skills, food science and food safety, as well as food consumption and waste practices. In a recent review, six themes related to FL were identified: skills and behaviors, food/health choices, culture, knowledge, emotions, and food systems (Truman, Lane, & Elliott, 2017). Growing recognition of the importance of nutrition and FL in promoting optimal health outcomes is underscored by the emergence of literature that assesses these constructs across various adult and pediatric populations (Ahire, Shukla, Gattani, Singh, & Singh, 2013; Cullerton, Vidgen, & Gallegos, 2012; Gibbs & Chapman-Novakofski, 2013; Yin et al., 2012).

Although robust literature reviews and critically appraises existing measures of HL and their psychometric properties (Jordan, Osborne, & Buchbinder, 2011), no such appraisal of available tools measuring NL and FL currently exists. Given the increased focus on NL and FL, a critical examination of the range of currently available measures will help ensure the generation of credible data to inform clinical practice, intervention development, and health policies. Advancement of the field also hinges on the use of measures that demonstrate sound validity (extent to which the tool measures what it purports to measure) and reliability (extent to which resultant scores are reproducible and free from error). The aim of this current review was to assess the psychometric properties and scope of currently available measures of NL or FL. It also assessed the extent to which measures capture constituent elements of their intended constructs.

## Methods

The review was planned and conducted using PRISMA (Preferred Reporting Items for Systematic Reviews and Meta-Analyses) guidelines (Moher, Liberati, Tetzlaff, & Altman, 2009). A systematic search of CINAHL, MEDLINE, EMBASE, ERIC, PubMed, Scopus, PsycInfo, Cochrane Database Library, Global Health, and Dissertations Abstracts International was performed using Boolean search terms from the inception of the databases until January 2016. No limitations were placed on language or publication type. Follow-up searches were conducted in July 2016, January 2017, and January 2018. Reference lists of retrieved articles were also screened for relevant studies.

The search terms included: food* or nutrition* or cook* or diet* AND “health literac*” or literac* or readability or “reading level*” or “media literac*” or “information literac*.” The same search terms were used for all databases.

### Inclusion Criteria

Publications were included if (1) the article reported on original research that included an instrument or method used to measure NL or FL; (2) assessed an adult population; and (3) was written in English. Gray literature (e.g., theses, dissertations) was included. Studies were excluded if they included instruments that were direct translations of the original version, or were published in languages other than English due to language barriers, or were designed for children or adolescents, as NL for minors is largely influenced by parents/guardians.

### Analytic Approach

Identified measures were evaluated for purpose, scope, face validity, content validity, construct validity, reliability, responsiveness to change over time, and generalizability using a modified version of a framework developed for the critical appraisal of health assessment tools (Jolles, Buchbinder, & Beaton, 2005), which has been used to evaluate HL measures (Jordan et al., 2011). Retrieved measures were also reviewed for domains of NL and FL as identified in existing reviews of definitions and conceptual models (Krause et al., 2016; Krause et al., 2018b; Velardo, 2015). The domains included components of functional, interactive, and critical literacy, as identified in Nutbeam's (2000) definition of HL, and adapted for a nutrition and FL context by Velardo (2015) and Krause et al. (2016). Instrument characteristics were abstracted and independently appraised by two reviewers, with disagreements resolved through discussion to consensus.

## Results

### Characteristics of Retrieved Measures

**Figure 1.**
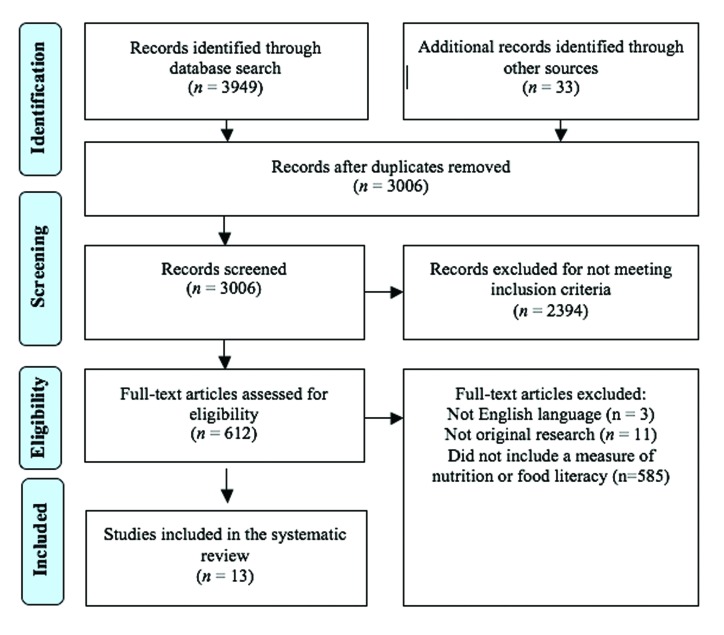
PRISMA (Preferred Reporting Items for Systematic Reviews and Meta-Analyses) flow chart showing results of systematic review of nutrition literacy measures.

Results of the search process are summarized in the PRISMA flow chart (**Figure 1**). Thirteen instruments met the inclusion criteria (Aihara & Minai, 2011; Chau et al., 2015; Coffman & La-Rocque, 2012; Diamond, 2007; Gibbs et al., 2016a; Gibbs et al., 2016b; Gibbs, Camargo, Owens, Gajewski, & Cupertino, 2017a; Gibbs, Ellerbeck, Gajewski, Zhang, & Sullivan, 2018; Guttersrud, Dalane, & Pettersen, 2014; Krause, Beer-Borst, Sommerhalder, Hayoz, & Abel, 2018a; Palumbo et al., 2017; Ringland, Gifford, Denyer, & Thai, 2016; Weiss et al., 2005). Seven were developed in the US (Coffman & La-Rocque, 2012; Diamond, 2007; Gibbs et al., 2016a; Gibbs et al., 2016b; Gibbs et al., 2017a; Gibbs et al., 2018; Weiss et al., 2005); the remaining six were developed in Australia (Ringland et al., 2016), Switzerland (Krausse et al., 2018a), Norway (Guttersrud et al., 2014), Hong Kong (Chau et al., 2015), Italy (Palumbo et al., 2017), and Japan (Aihara & Minai, 2011).

Eight of the 13 instruments purportedly measured NL (Aihara & Minai, 2011; Coffman & La-Rocque, 2012; Diamond, 2007; Gibbs et al., 2016a; Gibbs et al. 2016b; Gibbs et al., 2017a; Owens, 2015; Ringland et al., 2016), and two assessed FL (Krause et al., 2018a; Palumbo et al., 2017). Two instruments assessed food label literacy—the “Electronic Nutrition Literacy Tool” (Ringland et al., 2016) and the Newest Vital Sign (NVS), although the latter of the two was designed as a brief screening tool to assess limited literacy within the health care setting (Weiss et al., 2005). Because the NVS-Spanish was a direct translation of the NVS (Weiss et al., 2005), it was excluded. One measure assessed HL related to salt intake (Chau et al., 2015) and was included in the current review given its nutrition-related HL focus. Although one study (Mearns, Chepulis, Britnell, & Skinner, 2017) purported to use a measure of NL, closer inspection revealed that the measure was originally designed to assess nutrition knowledge (Chepulis & Mearns, 2015), rather than NL per se, and thus, was excluded from the review.

Eight instruments directly assessed NL (Aihara & Minai, 2011; Diamond, 2007; Gibbs & Chapman-Novakofski, 2013; Guttersrud et al., 2014; Krause et al., 2018b; Ringland et al., 2016; Weiss et al., 2005), or FL (Krause et al., 2018a; Palumbo et al., 2017) abilities. Four instruments were derivatives of NL instruments. One instrument was derived from the Nutrition Literacy Scale (NLS; Gibbs & Chapman-Novakofski, 2013) and adapted for a Spanish-speaking population (Coffman & La-Rocque, 2012). The other three derivatives were adaptations of the Nutrition Literacy Assessment Instrument (NLit; Gibbs & Chapman-Novakofski, 2013) breast cancer patient (Gibbs et al., 2016a), a Spanish-speaking Latino (Owens, 2015), and parent populations (Gibbs et al., 2016b).

### Reliability, Validity & Generalizability

**Table 1** summarizes the characteristics of included instruments including their psychometric properties. **Table 2** enumerates domains identified in each measure. Below we describe each measure and the results of our critical appraisal.

### Nutrition Literacy Scale

The NLS (Diamond, 2007) was developed in the US as a research tool to assess comprehension of nutritional information. The NLS was modeled on the reading comprehension section of the widely-used Short Test of Functional HL in Adults (S-TOFHLA; Baker, Williams, Parker, Gazmararian, & Nurss, 1999), which assesses reading, writing, and numeracy in health care settings. Like the S-TOFHLA, the 28-item NLS uses the modified Cloze procedure, in which a word is excluded from a sentence and respondents are asked to identify the correct response from four options. Item development was guided by sentences found in nutrition-related websites, including the Mayo Clinic's Food and Nutrition Center, Tufts' Nutrition Navigator, and the United States Department of Agriculture Center for Nutrition Policy and Promotion, as cited in Diamond (2007). NLS scores range from 2 to 28, with no numeric criteria specified to distinguish between inadequate and adequate NL.

The original 21-item NLS was pilot-tested in a sample of 132 adults including family medicine patients, local university students, municipal employees, and community members. The revised 22-item scale reflected reading comprehension rather than nutrition knowledge, and was further tested in a sample of 103 family medicine patients (Diamond, 2007), lengthened to 32 items, and subsequently shortened to 28 items after item analyses. Details on item revisions were not provided.

Psychometric assessment of the NLS was conducted with adult patients recruited from family medicine practices (*n* = 341). Internal consistency reliability, assessed using Cronbach's alpha, was 0.84. Construct validity was estimated by comparing NLS scores to S-TOFHLA scores, demonstrating a moderately strong relationship between the two scales (Pearson correlation coefficient = 0.61). Responsiveness to change over time was not assessed. The NLS has since been used in various populations including adolescents (D'Amato-Kubiet, 2013), middle-aged men (Duncan et al, 2014); the elderly (age 65+ years; Patel et al., 2013), weight-reduction program participants in Norway (Borge, 2012), and primary health care employees in Brazil (Sampaio et al., 2014).

### Spanish Nutrition Literacy Scale

The Spanish NLS (Coffman & La-Rocque, 2012) was developed from the NLS to assess NL in Spanish-speaking Latino adults in the US. Modifications for both content and language were made, resulting in the exclusion of one item due to translation issues and the addition of three items related to obesity and weight management. Although no cut-off scores were specified, higher scores denote greater NL.

Psychometric assessment of the Spanish NLS was performed with a Latino population from the southeastern US (*n* = 134). Items were assessed for meaning, relevance, and clarity through one 2-hour focus group with participants recruited from a Latino Service Agency; suggestions for alternative wording were sought from participants for items that were unclear. The scale yielded a reliability score of 0.95 using the Kuder-Richardson coefficient of reliability (KR-20). Regarding construct validity, the Spanish NLS was moderately associated with the S-TOFHLA (Spearman's rank correlation coefficient = 0.65, *p* < .001), and weakly associated with NVS (Spearman's rho = .16, *p* = .08). Responsiveness to change over time was not assessed.

### Nutrition Literacy Assessment Instrument

The NLit (Gibbs & Chapman-Novakofski et al., 2013; Gibbs, 2012; Gibbs et al., 2018; Gibbs et al., 2017b) was originally developed to assess NL within a nutrition education setting in the US.

Initial item development was based on a literature review and panel discussion with nutrition education experts to identify basic skills needed to understand nutrition/diet information. The original 40-item scale was reviewed by 135 registered dieticians for content validity, and pilot-tested with 26 people attending nutrition education consultations with registered dieticians (Gibbs & Chapman-Novakofski, 2013).

The item pool was subsequently expanded to 71 items; the methods used to generate new items were not provided. Content review by nutrition education and survey development experts, and cognitive interviews with 12 primary care patients, resulted in 66 items across 6 domains using different measurement procedures: 2 domains use the cloze procedure (Nutrition and Health; Energy Sources in Food); 3 domains use multiple response options (Household Food Measurement, Food Label and Numeracy, Consumer Skills); and the last domain asks respondents to categorize foods into the correct grouping (Food Groups) (Gibbs et al., 2017b). Psychometric assessment of the 66-item measure was performed on a sample of adults (*n* = 429) with nutrition-related chronic disease (e.g., diabetes, hyperlipidemia, hypertension, overweight/obesity) (Gibbs et al., 2018). Construct validity was assessed using binary confirmatory factor analysis (CFA), yielding a comparative fit index (CFI) of ≥0.90 and a root mean square error of approximation (RMSEA) of ≤0.06, indicating an acceptable fit of the data to the model.

Two versions of the tool were then created—a long form (64 item) and a short-form (42 item). CFI (0.975) and RMSEA (0.02) indices for the full 64-item scale demonstrated good model fit. Each domain also showed good model fit, as demonstrated by acceptable CFI and RMSEA indices (≥0.90 and ≤0.06, respectively), except for the food groups domain (CFI = 0.875). Reliability was assessed using a CFA-based measure, entire reliability (Alonso, Laenen, Molenberghs, Geys, & Vangeneugden, 2010), which was high for both the 64-item instrument (0.97; 95% confidence interval [CI], [0.96, 0.98]), and each domain (range, 0.75–0.95). Overall, test-retest reliability was also adequate for the full scale (Pearson's correlation coefficient = .88; 95% CI [0.86, 0.90]); however, good to adequate test-retest reliability was found for only 2 of the 6 domains (Food Label and Numeracy, Energy Sources in Food). The intraclass correlation coefficient (ICC), a measure of stability over time, was strong for the 64-item measure (0.88), but poor to adequate across the 6 domains (range, 0.5–0.8). Items with low reliability and low item to domain correlation were omitted from the 64-item instrument to create the 42-item NLit (CFI = 1; RMSEA = 0); model fit was good for the six domains as well (CFI ≥0.90; RMSEA ≤0.06). Entire reliability was high for both the 42-item instrument (0.96; 95% CI [0.95, 0.96]) and each domain (range 0.75–0.94). Overall test-retest reliability for the full scale was adequate (*r* = .88; 95% CI [.85, .90]), but ranged from poor (*r* = .43) to adequate (*r* = .76) across domains.

Scoring thresholds were determined by comparing the linear relationship between the 64-item NLit and the Healthy Eating Index (HEI-2010) scores (e.g., 64-item NLit scores ≤44 were associated with the lowest quintile of HEI-2010 scores), with the developers suggesting three scoring categories for both the 64-item and 42-item instruments: likelihood of poor NL (≤44/≤28); possibility of poor NL (45-57/29-38); and possibility of good NL (≥58/≥39).

### Nutrition Literacy Assessment Instrument for Breast Cancer

The 64-item NLit for Breast Cancer (NLit-BCa; Gibbs et al., 2016a) adapted the NLit for administration in primary and secondary breast cancer prevention populations to include concepts from the American Cancer Society's diet and cancer prevention guidelines (Kushi, Byers, Doyle, & Bandera, 2006). The NLit-BCa is comprised of 9 to 15 items across six domains, including 1 domain assessing consumer food-shopping skills whose development is not described. Interpretation of the scores is unclear; however, one study using the NLit-BCa assigned 1 point for each correct answer and calculated weighted percentages to give each domain equal distribution in the total score (Parekh et al., 2017).

The NLit-BCa was reviewed by three cancer nutrition experts for content by assessing the relevance of items in each domain, clarity, and potential redundancy. Modified items for the NLit-BCa then underwent cognitive testing with breast cancer survivors (*n* = 18) to assess whether items were understood as intended. Although composite reliability scores were acceptable for four domains (>0.7; Food Label & Numeracy: 0.87; Macronutrients: 0.77; Food Groups: 0.95; Consumer Skills: 0.84), two domains had scores lower than the accepted minimum (<0.7; Nutrition and Health: 0.54; Household Food Measurements: 0.65). The NLit-BCa was also administered 4 weeks apart to 30 breast cancer patients in remission, and 17 women at high risk for breast cancer to assess test-retest reliability. One domain exhibited substantial test-retest reliability (Food Label and Numeracy: 0.87); two domains showed moderate reliability levels (Nutrition and Health: 0.68; Macronutrients: 0.71), and the remaining three domains demonstrated fair reliabilities (Household Food Measurements: 0.44; Food Groups: 0.47; Consumer Skills: 0.49).

Construct validity was ascertained via CFA and convergent validity assessment. CFI indices ranged from 0.506 to 1 and RMSEA ranged from 0 to 0.601 across domains; only three domains (Food Label and Numeracy; Food Groups; Consumer Skills) showed good model fit by acceptable CFI (≥0.90) and RMSEA (≤0.06). The six NLit-BCa domains were compared with diet quality (HEI-2010) to gauge convergent validity. Five domains (Macronutrients, Household Food Measurement, Food Label and Numeracy, Food Groups, and Consumer Skills) showed significant positive relationships with diet quality (*p* < .05). The domain Nutrition and Health was not significantly associated with diet quality. Neither test-retest reliability nor responsiveness to change over time were assessed. The NLit-BCa has limited generalizability, having only been used in samples of women at high risk of breast cancer (*n* = 17) and breast cancer survivors in the rural Midwest (*n* = 55) and Eastern seaboard (*n* = 59) of the the US.

### NL Assessment Instrument-Spanish

Derived from the NLit, the Spanish version NLit-S was developed through a rigorous translation and adaptation process. First, the research team conducted a review of the items to assess relevance to the target population, replacing several food items with more widely recognized items. The instrument was independently translated by two native Spanish speakers and distributed to three other native Spanish speakers for review and revision of the translations; the latter three also decided on adaptations for inclusion. Three nutrition education professionals with expertise on the target population reviewed items for content validity. Cognitive interviews were conducted with three native Spanish speakers to assess language clarity and familiarity with food items.

Both CFA and convergent validity assessment were employed to gauge construct validity. CFI (>0.90) and RMSEA (<0.08) indices demonstrated acceptable model fit for the total scale and for each domain. Total scale NLit-S scores positively correlated with Short Assessment of Health Literacy-Spanish (SAHL-S) scores (*r* = .521, *p* < .001), which is a measure of HL for Latinos in Spanish (Lee, Stucky, Lee, Rozier, & Bender, 2010). Except for the Household Food Measurement domain, all NLit-S domains correlated with SAHL-S scores. The reported entire reliability was high for the total scale (0.99) and each domain (0.89–0.97). Cronbach's alpha for the total scale was good (0.92), however, alpha-levels for the individual domains ranged from 0.61 to 0.86, with three domains yielding an alpha below 0.70 (Nutrition and Health, Household Food Measurement, Consumer Skills). The NLit-S has limited generalizability, having been created for a Midwestern US Spanish-speaking Latino population.

### Nutrition Literacy Assessment Instrument–Parents

The 42-item NLit Parents (NLit-P) is a modified and shortened version of the NLit reflecting content and food items relevant for parents of preschoolers (age 4–6 years), as determined by two registered dieticians. The NLit-P was comprised of five domains: Nutrition & Health, Household Food Measurement, Food Label & Numeracy, Food Groups, and Consumer Skills. Construct validity, as assessed using CFA, demonstrated good model fit for 4 of the 5 domains; CFI and RMSEA indices for the Nutrition and Health domain were 0.581 and 0.1, respectively. Evaluation of the tool's concurrent validity found significant positive relationships between NLit-P scores and child diet quality (*r* = .418, *p* <0.001), income (*r* = .477, *p* <.001), parental age (*r* = .398, *p* < .001), and parental education (*r* = .595, *p* < .001); an inverse relationship was found between parental NL and parent BMI (*r* = −.306, *p* = .002). Entire reliability across the five domains was varied, with two domains demonstrating adequate reliability (Nutrition & Health: 0.84; Food Groups: 0.85), one domain demonstrating moderate reliability (Food Label & Numeracy: 0.78), and two domains showing questionable reliability (Household Food Measurement: 0.47; Consumer Skills: 0.55). Test-retest reliability was not assessed.

### Newest Vital Sign

Although developed by an expert panel in the US to assess HL concepts and general literacy within the primary care setting, the NVS (Weiss et al., 2005) is comprised of six items related to a nutrition label. Five candidate scenarios and representative items were initially proposed and refined after feedback from patients, interviewers, and data analysts on clarity and ease of scoring. The final short form uses a single nutrition-related scenario and evaluates the ability to use numbers and mathematical concepts (numeracy). Correct answers are given 1 point, with summed scores indicative of varying levels of literacy (>4 adequate literacy; 2–4 possibility of limited literacy, and <2 greater than 50% chance of having marginal/inadequate literacy).

Initial assessment of the NVS showed adequate internal consistency reliability (Cronbach's alpha = 0.76; Weiss et al., 2005). Across multiple studies, moderate associations have been found between the NVS and the TOFHLA (Pearson correlation coefficient [*r*] = .59–.62; Weiss et al., 2005; Wolf, Curtis, Wilson, & Revelle, 2012), the S-TOFHLA (Pearson's *r* = .54-.62; Kirk, et al. 2012; Osborn, et al., 2007), and the Rapid Estimate of Adult Literacy in Medicine (Pearson's *r* = .47), demonstrating moderate levels of construct validity (Wolf et al., 2012). Test-retest reliability or responsiveness to change over time has not been reported. A recent review highlights the measure's generalizability (Shealy & Threatt, 2016) across a range of adult ethnic populations, (e.g., White, Black, Hispanic/Latino), medical conditions (e.g., people with diabetes and their caregivers, kidney transplant candidates), and contexts (e.g., pregnancy, lactation).

### Electronic-Nutrition Literacy Tool

The 12-item Electronic-Nutrition Literacy Tool (e-NutLit; Ringland et al., 2016) was developed in Australia to assess food label literacy in adults. Four key domains were identified through examination of the extant literature and focus groups with dieticians and included Nutrition Information, Calculating/Converting Serving Sizes, Comparing Products Using Nutrition Information Labels, and Influence of Endorsement Labels. Twelve items were added to gauge exposure to label reading, including food shopping responsibility, reported frequency of food label reading, and engagement in diet modification in response to a medical condition, as well as demographic information. A composite score is created by summing correct responses, with higher values indicating higher levels of NL.

Content validity, assessed by way of item comprehension, was determined through cognitive interviews with participants with low to intermediate HL (an NVS score below 4 of 6); however, these results have not been reported. Neither internal consistency reliability, test-retest reliability, nor responsiveness to change have been reported. However, the e-NutLit's construct validity was tested and a significant positive association was found with the NVS (Spearman's rho = .73, *p* < .001). The e-NutLit has limited generalizability, having only been assessed in university obesity clinic patients and final year dietetic students (*n* = 61).

### Short Food Literacy Questionnaire

The 12-item Short Food Literacy Questionnaire (SFLQ; Krause et al., 2018a) was originally developed in Switzerland as part of an intervention study to reduce salt consumption among Swiss workers (Krause et al., 2016). A three-stage process, beginning with an examination of the extant literature to develop a working definition of the construct of interest (initially referred to as nutrition-specific HL) and the identification of relevant NL and HL measures for adaptation was employed in the tool's creation. The working definition included 12 nutrition-specific HL themes across three forms of HL (functional, interactive, critical). Items from existing nutrition and HL instruments were enumerated and assigned to nutrition-specific HL themes; new items were generated for themes without suitable items. The item pool underwent initial expert review to assess face validity, cognitive interviews with administrative and university employees (*n* = 12), and a survey of health sciences students (*n* = 63). The 12-item measure employed a Likert-type scale; individual item scores were summed to create a composite score (52 maximum) with no interpretation provided. Exploratory factor analysis identified a unidimensional structure.

Construct validity was assessed by examining associations with HL, nutrition knowledge, gender, and education. SFLQ scores showed a moderate correlation with European Health Literacy Survey (HLS-EU) scores (Sørensen et al., 2013; Spearman's rank correlation coefficient = 0.46). No differences were found between SFLQ scores and correct responses to a single item related to composition of a healthy plate (Wilcoxon rank sum test [*Z*] = 1.68, *p* = .09). However, higher SFLQ scores were associated with correct response to recommended amount of daily salt consumption (*Z* = 3.93, *p* <.001). Women had a higher SFLQ score compared to men, but no association was found between SFLQ scores and education level. Cronbach's alpha for the 12-item scale was 0.82. Responsiveness to change over time was not reported. The measure has only been administered to employed, German-speaking Swiss people between the ages of 15 and 65 years.

### Critical Nutrition Literacy Instrument

The Critical Nutrition Literacy (CNL) instrument was developed in Norway to assess nursing students' CNL. The authors define CNL as “being proficient in critically analyzing nutrition information and advice, as well as having the will to participate in actions to address nutritional barriers in personal, social and global perspectives” (Guttersud et al., 2014). The 19-item instrument employs 5-point Likert-type scales (*disagree strongly* to *agree strongly*) to assess two domains of CNL: “engagement in dietary habits” (8 items), and “taking a critical stance towards nutrition claims and their sources” (11 items). Further details on the development of the scales were not available in English.

Results of a Rasch analysis (Rasch, 1960) conducted to assess construct validity revealed disordered thresholds for 8 items on the “claims” scale and the response options revised to a 4-point Likert-scale. One item from the “claims” scale underdiscriminated (neither stratified nor measured) per item fit residuals and chi-square statistics, and therefore was discarded. Another item showed uniform differential item functioning (item assessed different abilities for members of individual subgroups, such as gender) and underwent the “person factor split” procedure. Rephrasing of problematic items has been recommended prior to further field trials (Guttersrud et al., 2014). The instrument has shown adequate internal consistency reliability (engagement scale: alpha = 0.80; claims scale: alpha = 0.70 with one item deleted). Test-retest reliability or responsiveness to change has not been assessed. The measure has only been used with Norwegian nursing students.

### Health Literacy Scale for Low Salt Consumption–Hong Kong Population

The Health Literacy Scale for Low Salt Consumption–Hong Kong Population (CHLSalt-HK; Chau et al., 2015) was developed to assess sodium intake in residents of Hong Kong. Sodium intake in Hong Kong greatly exceeds the level recommended by the World Health Organization. Assessing HL related to salt consumption among older adults could guide the development of interventions that target their knowledge gaps, misconceptions, or poor dietary practices. The 49-item CHLSalt-HK was based on three domains of HL (Functional Literacy, Factual and Procedural Knowledge, and Awareness) identified by Frisch, Camerini, Diviani, and Schulz (2012). Item development was guided by prior literature on knowledge, attitudes and dietary practices related to salt consumption, and nutrition label reading. Eight broad areas were included in the scale: (1) functional literacy (term recognition and nutrition label reading; 3 items), (2) knowledge of the salt content of foods (13 items), (3) knowledge of the diseases related to high salt intake (8 items), (4) knowledge of international standards (2 items), (5) myths about salt intake (4 items), (6) attitudes toward salt intake (7 items), (7) salty food consumption practices (9 items), and (8) nutrition label reading practices (3 items). Response options included either a 5-point Likert scale (item score of 0–2) or 4 multiple choice options (item score of 0 or 2). The total scale score ranges from 0 to 98, with higher scores indicating higher HL related to salt intake. The scale reportedly takes 10 to 15 minutes to complete.

Content validity was assessed by a panel of eight experts including doctors, nurses, and dietitians. The item level content validity index (CVI) for the CHLSalt-HK ranged from 0.86 to8 1.00, with a scale level CVI of 0.99, suggesting adequate content validity (CVI ≥0.78). After expert review, the revised item pool was piloted with 17 elderly adults to assess readability and interpretation of items.

Construct validity was assessed through CFA and convergent validity assessment in a sample of 603 Cantonese-speaking adults age 65 years or older. The initial factor structure with 54 items across eight first-order factors, and one second-order factor (HL related to low salt intake) did not yield adequate model fit (Rapid Estimate of Adult Literacy in Medicine [RMSEA] = 0.03; standardized root mean square residuals [SRMR] = 0.09; CFI = 0.87); thus, items with insignificant or poor loading (<0.2) were removed, leaving 49 items. The final model showed adequate model fit (RMSEA = 0.03; SRMR = 0.09; CFI = 0.90). Convergent validity was assessed through correlation analysis between the CHLSalt-HK and Chinese Health Literacy Scale for Chronic Care (CHLCC; Leung et al., 2013), a measure developed to assess the HL of patients with chronic disease. Although low correlation between the two scales was expected given the different focus of each measure, the CHLSalt-HK and CHLCC were significantly correlated (Pearson correlation coefficient *r* = .29; *p* < .001), thus convergent validity was not supported.

Discriminant validity was assessed through examining differences in CHLSalt-HK scores between those with and without hypertension, and those who were and those who were not aware of the public education slogan about nutrition labels and sodium intake. People without hypertension yielded a significantly higher CHLSalt-HK score (by 1.844 points) than people with hypertension (95% CI [0.11, 3.58]); a very small effect size [Cohen's *d* = .171]). In addition, people who had heard of the public health slogan scored significantly higher by 3.928 points (95% CI [1.74, 6.12]) compared to those who had not heard of the slogan, which supported adequate discriminant validity.

Internal consistency for the total scale, as assessed using Cronbach's alpha, was 0.80, suggesting adequate internal consistency; however, Cronbach's alpha across the eight factors ranged from poor to adequate (0.39–0.86). Test-retest reliability over a 2-week period (*n* = 41) was adequate (intraclass correlation coefficient [ICC] = .85; 95% CI [.707, .919]). Although inter-rater reliability assessment was examined through self-administration and face-to-face interview (*n* = 38), inconclusive results were reported (ICC = 0.70; 95% CI [.457, .839]) due to the wide confidence interval. Responsiveness to change has not been assessed. The CHLSalt-HK has only been used with older Chinese adults (age 65 years and older), which limits generalizability.

### Italian Food Literacy Survey

The Italian Food Literacy Survey (IT-FLS; Palumbo et al., 2017) was developed to assess individual food literacy skills in Italy. A concept validation approach (SØrensen et al., 2013) was used to design the 47-item survey. Vidgen and Gallegos' (2014) definition and conceptual model of FL was used to guide survey development, with their four conceptual domains aggregated to three domains for inclusion in the survey: (1) ability to plan and manage food (16 items); (2) ability to select and choose food (15 items), and; (3) ability to prepare and consume food (16 items). Item generation and refinement was guided by panel discussion with 12 experts including dietitians, primary care providers, and scholars in HL and FL using the Delphi procedure. Face validity was assessed through a focus group comprised of 15 dieticians. The draft survey was pretested on a sample of 60 Italian citizens to assess item comprehension, with item-to-item analysis and principal component analysis (PCA) conducted to refine items. Items with low discriminative power, as determined through 95% or more answers in the same category, were removed. PCA was fixed at three to reflect domains that guided survey development, with items that demonstrated low factor loadings (<0.30) or small difference on any two components removed. A 4-point Likert scale was used for response options (*very difficult* to *very easy*).

Internal consistency and convergent validity of the IT-FLS was assessed using data from a convenience sample of 158 adults. Internal consistency, assessed using Cronbach's alpha for the total scale (General Food Literacy Index; 0.91) and the three individual scales (Plan and Manage FL = 0.879; Select and Choose FL = 0.881; Prepare and Consume FL = 0.893) was adequate. Convergent validity as assessed through correlation analysis between IT-FLS and NVS scores, with the NVS showing positive and significant correlation (*p* < .01, 2-tailed) with the total score (.378) and the three individual scales (.327–.374). Scores for the IT-FLS ranged from 0 to 50, with the scoring criteria based on the HLS-EU survey (Sørensen et al., 2013; 0 to 25 = inadequate FL, 25.01 to 33 = problematic food literacy, 33.01 to 42 = sufficient food literacy, 42.01 to 50 = excellent food literacy). Test-retest reliability, further assessment of the final factor structure (e.g., using CFA methods), or responsiveness to change have not been reported.

### Nutrition Literacy Items for an Elderly Japanese Population

Aihara and Minai (2011) developed a 10-item measure to assess NL in an elderly Japanese population (age ≥75 years). Item development was guided by contents of the “Japanese Food Guide Spinning Top,” an illustrated nutritional chart, and the “Dietary Guidelines for Japanese” people (Japanese Ministry of Agriculture Forestry and Fisheries, 2005). The self-report tool assesses ability to obtain basic diet information and knowledge of recommended dietary habits with two response options (*I do/don't know*). Affirmative responses were assigned 1 point and summed to create a composite score, with 10 points indicative of adequate NL; any score under 10 indicates limited NL. Neither test-retest reliability, construct validity, content validity, nor responsiveness to change have been assessed; however, internal consistency reliability was 0.86.

## Discussion

The emergence of the concepts NL and FL have significantly enhanced our understanding of the complex array of factors contributing to person's capacity to make quality nutrition decisions and enact healthy dietary behaviors. To our knowledge, this is the first systematic review and critical appraisal of existing tools developed to assess these constructs. Notably, 11 of 13 measures purported to assess NL, rather than FL. Substantial variation in methodological rigor was observed across measures. For instance, only 3 of 13 measures were based on a working definition of NL or FL, and none assessed responsiveness to change over time. Further, only three instruments had been assessed for test-retest reliability, and only eight measures included directions for scoring to differentiate between various levels of literacy. Overall, the NLit had the strongest psychometric properties.

As noted above, only two measures purported to assess FL (Krause et al., 2018a; Palumbo et al., 2017), as distinct from NL, in an adult population. In a recent review of NL and FL definitions, Krause et al. (2018b) assert that definitions of FL more comprehensively capture the skills and competencies critical to a person's capacity to make quality food and nutrition decisions. They also argue that FL definitions better capture volitional and behavioral factors (e.g., awareness, attitudes, and motivations), such as food appreciation, motivation to prepare healthy meals, and perceptions of cooking and eating, that may influence a person's capacity to act on nutritional knowledge and skills. We concur with claims that, as compared to NL, FL is a broader and more appropriate concept for guiding the development of nutrition education strategies (Krause and Sommerhalder, 2016; Smith, 2009). Given the findings of this review, continued efforts are needed to develop psychometrically sound measures designed to assess FL and its key domains, including volitional and behavioral factors. Such efforts will facilitate the rigorous assessment of subsequent educational strategies and interventions.

Further, HL measurement researchers have argued that comprehensive assessment be built explicitly from a testable theory or conceptual framework to identify key elements for inclusion in measures (Pleasant, McKinney, & Rikard, 2011). However, only two instruments (Krause et al., 2018a; Palumbo et al., 2017) were guided by a conceptual model. Our analysis also revealed gaps in the assessment of broader domains captured in conceptualizations of NL, including the context in which NL capacities are developed and applied, such as past experiences, sociocultural norms, and structural factors that influence NL (Velardo, 2015). The opportunity for people to develop skills and capacities to engage with internal and external resources has also been highlighted (Velardo, 2015). Although the NLit, and its derivatives capture themes relevant to functional, interactive, and critical NL, including food measurement and consumer skills, and have shown adequate psychometric properties, it was unclear how its sixth domain (Consumer Food-Shopping Skills) was later derived. The lack of a guiding theoretical framework in combination with unmeasured domains of NL leave existing NL and FL measures particularly deficient in their ability to accurately identify gaps in people's capacities and specific areas for remediation.

Findings of seven studies (Chau et al., 2015; Coffman & La-Rocque, 2012; Diamond, 2007; Gibbs, Camargo, et al., 2017; Palumbo et al., 2017; Ringland et al., 2016; Weiss et al., 2005) that assessed construct validity of the NL instruments were obtained through comparisons with other HL measures, with mixed results. Not surprisingly, strong correlations were found between the S-TOFHLA and the NLS and its Spanish derivative, as the reading comprehension domain of the S-TOFHLA was used to guide development of these NL measures. The Spanish NLS, however, had low correlation with NVS, suggesting the possibility that Spanish NLS and NVS were assessing different underlying constructs. In contrast, the strong correlations between the NVS and the IT-FLS and e-NutLit, measures that assess FL or food label literacy, suggest that these instruments were assessing the same underlying construct. Construct validity of NL measures assessed through comparisons with existing measures of functional HL and food label literacy (e.g., NVS) may inadequately assess the broader domains reflected in NL definitions and conceptualizations, and thus may not be comparable. This criticism has also been noted in literature around HL instruments, in which criterion validity has been assessed through comparisons with functional literacy assessments that may inadequately capture the HL domains (Altin, Finke, Kautz-Freimuth, & Stock, 2014). To address these limitations, Gibbs et al. (2016a, 2016b, 2018) and colleagues assessed the convergent validity of three NL measures (NLit, NLit-BCa, and NLit-P) through comparisons with diet quality/child diet quality. Given that positive dietary practices have been identified as an ideal outcome of nutrition literacy (Velardo, 2015), assessing the convergent validity of NL measures in relation to available measures of diet quality and to competencies that measures purport to assess, such as nutrition knowledge and food skills, is recommended.

Indeed, there have been calls to move beyond assessment of functional NL to capture sociocultural domains that influence NL (Velardo, 2015). Within the field of HL, objective instruments that assess functional skills (e.g., reading, comprehension, and numeracy) have been criticized for their narrow content and focus on declarative knowledge (i.e., knowing facts or information), and consequently their inability to identify suboptimal skills and capacities (Jordan et al., 2011). In response to these criticisms, multidimensional measures have emerged that include subjective (i.e., self-report based) components that assess broader domains of HL, including procedural knowledge-related elements (i.e., skills to perform specific tasks), such as health information seeking, interaction with the health system, patient-provider relationships, communication with health care providers, and the capacity to understand, process, and use health information (Altin et al., 2014). Nine of 13 instruments included in the present review were objective (i.e., task-based) assessments of a person's capacities, whereas three instruments (Aihara & Minai, 2011; Krause et al., 2018a; Palumbo et al., 2017) were subjective. One instrument included both objective and subjective items; however, it was focused specifically on HL skills related to salt intake (Chau et al., 2015).

To advance the interrelated fields of NL and FL, measures combining both objective and subjective components are needed. Whereas existing measures largely focus on nutrition-related print and functional literacy, future tools should aim to also assess skills-based concepts as means of identifying day-to-day challenges to engaging in optimal dietary practices. Inclusion of items measuring interactive components, such as the cognitive, food-related, and interpersonal communication skills needed to interact and share information with others (Krause et al., 2018b) should also be prioritized, along with the complex skills, motivation, and confidence needed to navigate the food system (Velardo, 2015). Measures that combine both objective, task-based items together with subjective items, like those included in recently developed HL tools (Osborne, Batterham, Elsworth, Hawkins, & Buchbinder, 2013; Sørensen et al., 2013), have the potential to further our understanding and assessment of potentially modifiable factors that influence dietary practices.

## Limitations

Although this current review contributes important knowledge of existing measures of NL and FL, it is not without limitation. For instance, our search strategy attempted to exhaustively identify instruments related to NL and FL, but we may not have identified all available instruments. In addition, our appraisal of available instruments was limited to information published in the literature. Strengths of the study are use of a structured framework to critically appraise the psychometric properties of available measures of NL and our evaluation of the capacity of these instruments to assess representative domains of NL.

## Conclusion

Our review provides insights into the current state of food and nutrition measurement through critical appraisal of the development and psychometric properties of existing measures. Further research is needed to address gaps in measurement, including development of well-defined, theoretically grounded measures that assess broader domains relevant to NL and FL. Development of comprehensive NL and FL instruments is needed to inform the development of and to rigorously evaluate interventions that effectively respond to nutritional information needs of the populations, and to promote and enhance optimal dietary practices.

## Figures and Tables

**Table 1:** Summary of the Characteristics and Critical Appraisal of Included Measures

**Instrument Characteristics**	**Name of Instrument**												
	**NLS**	**Spanish NLS**	**NVS**	**NLit**	**NLit-BCa**	**NLit-S**	**NLit-P**	**CNLI**	**e-NutLiT**	**SFLQ**	**NLQ-JP**	**CHLSalt-HK**	**IT-FLS**
1. Author (year of publication)	Diamond (2007)	Coffman & La-Rocque (2012)	Weiss et al. (2005)	Gibbs & Harvey (2017)	Gibbs et al. (2016a)	Gibbs, Camargo et al. (2017)	Gibbs, Kennet et al. (2016)	Guttersrud et al. (2014)	Ringland et al. (2016)	Krause, Beer-Borst et al. (2018)	Aihara & Minai (2011)	Chau et al. (2015)	Palumbo et al. (2017)
2. Country of origin	US	US	US	US	US	US	US	Norway	Australia	Switzerland	Japan	Hong Kong	Italy
3. Stated expertise of developers	Not reported	Not reported	A panel of HL experts	Nutrition education research experts	Nutrition education research experts	Nutrition education research experts	Nutrition education research experts	Unclear (manuscripts not in English)	Literacy assessment experts	Not reported	Not reported	Not reported	Not reported
4. Stated purpose and population	To assess NL in adults	To measure NL in Spanish-speaking adults	Brief screening tool to identify limited literacy through assessing ability to read and apply information from a nutrition label among English-speaking primary care patients	To assess NL in nutrition education settings; primary care patients with nutrition-related chronic disease	To assess NL among primary and secondary breast cancer prevention populations	To assess NL in a Spanish-speaking Latino population	To assess parental NL	To assess critical nutrition literacy in nursing students	To assess food label literacy using an electronic assessment tool among Australian adults	To measure self-rated food literacy	To assess NL in Japanese population	To assess health literacy related to salt consumption in older Chinese adults in Hong Kong	To assess individual food literacy skills
5. Method of development: How was the instrument developed (or modified if more applicable)	S-TOFHLA was used as a model. Items were constructed from declara-tive sentences found in nutrition-related websites	The NLS was translated into Spanish. Content validity was assessed through a focus group of Latino adults. One item from the NLS was removed due to lack of meaning following translation, and three additional items were included related to obesity and weight management	Five scenarios developed by a panel ofHL experts. Scenarios and questions were refined after feedback from patients, interviewers, and data analysts about the clarity and ease of scoring of items	Literature review and expert interviews were used to identify five domains of NL for inclusion. A sixth domain was included after pilot-testing a modified item pool with breast cancer patients	The original 40-item NLit that addressed five domains was modified to include concepts from the American Cancer Society's Diet & Cancer Prevention guidelines. A sixth domain was included to assess consumer food shopping skills. To ensure internal consistency, a 75-item draft instrument was initially developed that represented the psychometric properties in breast cancer survivors, and women at high risk for breast cancer	NLit items reviewed by research team to determine relevance of food items for Spanish-speaking Latino community. NLit items were translated through consensus translation by two native Spanish speakers	NLit item pool reduced to 42 items to reflect content and food items relevant for parents of pre-schoolers, as determined by two registered dietitians	Unclear (articles not in English)	Item development and key elements of NL for inclusion in the instrument were informed by data collected through focus groups with dieticians	Three-stage process comprised of: acquisition of background materials; questionnaire development by adapting existing HL questions to be nutrition-specific; and two-stage pre-test	Items were developed to assess participants knowledge of contents of the “Japanese Food Guide Spinning Top”	Based on three domains of HL identified in the literature: functional literacy, factual and procedural knowledge, and awareness. Item development was guided by prior studies on knowledge, attitudes and dietary practices related to salt consumption and nutrition label reading	A concept validation approach was used to design the survey, following the design and development of the European Health Literacy Survey (SØrensen et al., 2013). Three conceptual domains were identified based Vidgen and Gallegos' (2014) definition and model of FL
6. Number of items and domains	28 items; single domain	30 items; single domain based on knowledge of nutrition information	Six items based on five scenarios that varied in the type of literacy skills including reading comprehension and numeracy	Long-form (64-item) and short-form (42-item) versions represent six domains of NL: nutrition and health; energy sources in food; household food measurement; food label and numeracy; food groups; and consumer skills	64-item version of the NLit represented six domains of NL for breast cancer patients	64-item version of the NLit represented six domains of NL for Spanish-speaking Latino adults	42 items representing five domains: nutrition and health; household food measurement; food label and numeracy; food groups; and consumer skills	19 items; two domains: engagement in dietary habits (“engagement”); and taking a critical stance towards nutrition claims and their sources' (“claims”)	24 items; four domains: identifying nutrition information; calculating/converting serving sizes; comparing products using nutrition information panels; and influence of endorsement logos	16 items	10 items based on guidelines that assess recommended dietary habits	49 items; 8 domains: functional literacy (term recognition and nutrition label reading); knowledge of the salt content of foods; knowledge of the diseases related to high salt intake; knowledge of international standards; myths about salt intake; attitudes toward salt intake; salty food consumption practices; and nutrition label reading practices	47 items across three domains: plan and manage food literacy (16 items); select and choose FL (15 items); and prepare and consume FL (16 items)
7. How is it administered?	Interview, and pen and paper self-administration	Face-to-face interviews in Spanish	Interview administration	Pen and paper self-administration	Pen and paper self-administration	Pen and paper self-administration	Pen and paper self-administration	Pen and paper self-administration	Electronic tablet format with assistance from administrato	Pen and paper self-administration	Pen and paper self-administration	Pen and paper self-administration	Paper and pencil self-administraton
8. Special requirements for administration	Visual acuity sufficient to read instrument items	Near vision acuity of at least 20/200 on a handheld vision chart.	Visual acuity sufficient to readthe instruments being tested, and have grossly normalcognitive function	Speak and read in English, sufficient visual acuity to read instrument items	Speak and read in English, sufficient visual acuity to read instrument items	Speak and read in Spanish; sufficient visual acuity to read the instrument	Speak and read in English, sufficient visual acuity to read instrument items	Not reported	Visual acuity sufficient to read instrument items; capacity to use electronic tablet	Not reported	Sufficient Japanese reading skills	Speak and understand Cantonese	Speak and read Italian
9. Time it takes to administer	10 minutes	10 minutes	3 minutes	9 42-item) or 16 (64-item) minutes	Not reported	Not reported	Not reported	<20 minutes	Not reported	Not reported	Not reported	10–15 minutes	Not reported
10. Scoring													
10a. How is it scored	Single score, 0–28	0–30	Single score 0–6	Single score for each item	0–64. Weighted percentages are then calculated giving each domain equal distribution in the total score.	Single score for each item (0–64)	0–42. Weighted percentages are then calculated giving each domain equal distribution in the total score.	Not reported	0–12	Not reported	0–10	0–98	0–50
10b. Scoring categories	Poor (0–7), marginal (8–14), and adequate (15–28) NL	No scoring categories: a higher score indicatesmore NL and a lower score indicatesless NL	>4: adequate literacy when measured by the TOFHLA.A score <4 on the NVS, on the other hand, indicatesthe possibility of limited literacy. Clinicians shouldbe particularly careful in their communication with patients who score <2, as they have >50% chance of having marginal or inadequate literacyskills.	64 item: likelihood of poor NL (≤44); possibility of poor NL (45–57); likelihood of adequate NL (≥58) (42 item: likelihood of poor NL (≤28); possibility of poor NL (29–38); likelihood of adequate NL (≥39)	Not reported	Likelihood of poor NL (≤44); possibility of poor nutrition literacy.	Not reported	Not reported	Not reported	Not reported	Adequate (score = 10); in adequate (score <10)	Higher score indicates higher health literacy related to salt intake	Inadequate food literacy: 0–25; Problematic food literacy: 25.01–33; Sufficient food literacy: 33.01–42; Excellent food literacy: 42.01–50
**Critical appraisal of measures**													
1. Is the instrument based on an underlying conceptual framework?	No; the reading comprehension section of S-TOFHLA), was used as a model	No; based on NLS	No; based on scenarios that were developed by a panel ofHL experts based on the concepts and typesof scenarios used in HL research and ingeneral literacy	No; based on literature review and expert panel	No; based on NLit	No; based on NLit	No; based on NLit	Unclear (manuscripts not in English	No; focus groups findings with dieticians and literature	Yes; analyzed definitions of NL and FL and their core components in comparison to established concepts of HL	No; based on Japanese Food and Dietary-related guidelines	No; based on three domains of HL identified by Frisch et al. (2012).	Yes; based on a conceptual model of FL developed by Vidgen and Gallegos (2014)
2. Content validity													
2a. Were intended domains (i.e., relevant areas to be included and excluded) clearly stated?	No	Yes	Candidate scenarios that assess reading comprehension and numeracy	Yes	Yes	Yes	Yes	Yes	Yes	Yes	No	Yes	Yes
2b. Are all relevant components of each domain included?	Partial; the NLS assesses reading comprehension only	Yes	Yes	Yes	Yes	Yes	Yes	Yes	Unclear	No; 12 themes identified; however, included items only covered 10 themes	Unclear	Yes	Yes
3. Face validity													
3a. On the face of it does it describe the intended purpose	Yes	Yes	Yes	Yes	Yes	Yes	Yes	Yes	Yes	Yes	Yes	Yes	Yes
3b. Are the definitions of each category clearly specified?	Not applicable	Not applicable	Not applicable	Yes	Yes	Yes	Yes	Yes	Yes	Yes	Not applicable	Yes	Yes
3c. Are these definitions acceptable?	Not applicable	Not applicable	Not applicable	Yes	Yes	Yes	Yes	Yes	Unclear	Yes	Not applicable	Not applicable	Yes
3d. Are the methods for determining the presence and/or absence of criteria described and acceptable?	Yes	Not reported	Yes	Not reported	Not reported	Not reported	Not reported	Not reported	Yes	Not reported	Not reported	Not reported	Not reported
3e. Do the scoring categories sufficiently discriminate considering the stated purpose?	Not reported	Not applicable	Not reported	Yes	Not reported	Yes	Not reported	Not reported	Not reported	Yes	Not reported	Not reported	Yes
4. Construct validity: does the instrument perform in expected ways when compared with other NL/HL/literacy indices?	Partial; correlated with S-TOFHLA (Pearson correlation = .61). Low correlation with self-reported years of education (.41)	Partial; strong relationship with S-TOFHLA (Spearman's rank correlation co-efficient = .65, *p* < .001), and low correlation with NVS (Spearman's rank correlation coefficient = .16, *p* = .08)	Partial; moderate correlation with TOFHLA (Pearson correlation *r* = .59); S-TOFHLA (Pearson correlation coefficient *r* = .6). Low correlation with REALM (Pearson correlation coefficient *r* = .41)	Partial; five of six domains had a positive relationship with diet quality (as measured by HEI-2010; *p* < 0.05): (1) nutrition and health; (2) energy sources in food; (3) food label and numeracy; (4) food groups; (5) consumer skills	Partial; five of six domains had a positive relationship with diet quality (as measured by HEI-2010; *p* < .05): (1) macronutrients; (2) household food measurement; (3) food label numeracy; (4) food groups; and (5) consumer skills	Partial; five of six domains positively correlated with SAHL-S scores (r = 0.521, *p* < .001)	Partial; NLit-P scores had positive relationship with child diet quality (*r* = .418, *p* < 0.001), parental education (*r* = .595, *p* < 0.001), child diet quality (*r* = .418,*p* < 0.001), income (*r* = 0.477, *p* < .001), and parental age (*r* = .398, *p* < 0.001)	No comparisons conducted	Partial; positive relationship between eNutLit and NVS (Spearman's rank correlation coefficient = .73, *p* < .001). Among obese patients, eNutLit scores associated with education levels (*p* < .04) and food shopping responsibility (*p* < .0048)	No comparisons conducted	No comparisons conducted	No; although low correlation was expected between the CHLSalt-HK and the Chinese Health Literacy Scale for Chronic Care (Leung et al., 2013) given the different focus of each measure, the two scales significantly correlated (Pearson correlation coefficient = .29; *p* < .001)	Partial; positive and significant correlation (*p* < 0.01, two-tailed) found between NVS and IT-FLS total score (.378), and the three individual scales (.327 to .374)
5. Has sensitivity to change been demonstrated?	Not reported	Not reported	Not reported	Not reported	Not reported	Not reported	Not reported	Not reported	Not reported	Not reported	Not reported	Not reported	Not reported
6. Reliability													
6a. Has reliability been measured?	Not reported	Not reported	Not reported	Yes; test-retest reliability ranged between 0.51 and 0.80 across six domains; total scale test-retest reliability was adequate (0.88).	Yes; test-retest reliability ranged between fair and substantial across six domains (0.44–0.90).	Not reported	Not reported	Not reported	Not reported	Not reported	Not reported	Yes; test-retest reliability (*n* = 41) was adequate (ICC = 0.85; 95% CI [0.707, 0.919])	Not reported
6b. Inter-rater reliability	Not reported	Not reported	Not reported	Not reported	Not reported	Not reported	Not reported	Not reported	Not reported	Not reported	Not reported	Inconclusive. Comparison of self-administration vs. face-to-face interview (*n* = 38) was inconclusive due to wide confidence interval (ICC = 0.700; 95% CI [0.457, 0.839])	Not reported
6c. Intra-rater reliability	Not reported	Not reported	Not reported	Not reported	Not reported	Not reported	Not reported	Not reported	Not reported	Not reported	Not reported	Not reported	Not reported
6d. Internal consistency	Cronbach's alpha = .84	The Kuder-Richardson coefficientof reliability (KR-20), a variant of the Cronbach'salpha designed for scales with binary items, washigh (KR-20, 5.95), suggesting robust reliability.	Cronbach's alpha = .76	Entire reliability for the total scale was 0.97 (95% CI, [0.96, 0.98]). Entire reliability was >0.80 for five domains; one domain (consumer skills) approached acceptable reliability (0.75)	Entire reliability was >0.80 for three domains (food label & numeracy; food groups; and consumer skills). The remaining three domains approached acceptable reliability (0.54–0.77)	Entire reliability: total scale: 0.99; across six domains: 0.89–97 Cronbach's alpha: entire instrument: .91; across six domains: .61 to .84	Entire reliability across five domains ranged from 0.47–0.85	Engagement scale: Cronbach's alpha = .80; PSI = .77 Claims scale: Cronbach's alpha = .70 (one item deleted); PSI = .71	Not reported	Not reported	Cronbach's alpha = .86	Total scale: Cronbach's alpha = .86 Across eight domains: Cronbach's alpha = .39 to .86	Cronbach's alpha for the total scale (General Food Literacy Index; .91) and the three individual scales (plan and manage FL = .879; select and choose FL = .881; prepare and consume FL = .893)
7. Feasibility													
7a. Is it simple to understand?	Yes	Yes	Yes	Yes	Yes	Yes	Yes	Yes	Unclear	Yes	Yes	Yes	Yes
7b. Is it easy to perform and administer?	Yes	Yes	Yes	Yes	Yes	Yes	Yes	Yes	Yes	Yes	Yes	Yes	Yes
8. Domains covered in the measure	Knowledge of consumer-related topics in nutrition	Knowledge of consumer-related topics in nutrition	Ability to understand text, and reading and numeracy	Six domains: (1) nutrition and health; (2) energy sources in food; (3) household food measurement; (4) food label and numeracy; (5) food groups; (6) consumer skills	Six domains: (1) nutrition and health; (2) macronutrients; (3)household food measurement; (4) food label and numeracy; (5) food groups; (6) and consumer skills	Six domains: (1) nutrition and health; (2) macronutrients; (3) household food measurement; (4) food label and numeracy; (5) food groups; and (6)consumer skills	Five domains: (1) nutrition and health; (2) household food measurement; (3) food label and numeracy; (4) food groups; and (5)consumer skills	Two domains of CNL: (1)engagement in dietary habits (“engagement”); (2) taking a critical stance towards nutrition claims and their sources' (“claims”)	Four domains: (1)identifying nutrition information; (2) calculating/converting serving sizes; (3)comparing products using nutrition information panels; (4)influence of endorsement logos	Ten themes: across three forms of HL (functional, interactive, and critical)	Ability to obtain basic diet information about recommended dietary habits	Eight domains: (1) functional literacy; (2) knowledge of the salt content of foods; (3) knowledge of the diseases related to high salt intake; (4) knowledge of international standards; (5) myths about salt intake; (6) attitudes toward salt intake; (7) salty food consumption practices; and (8) nutrition label reading practices	Three scales: (1)plan and manage FL; (2) select and choose FL; (3) prepare and consume FL
9. Response options	Cloze procedure with four-option multiple choice format	Cloze procedure with four-option multiple choice format	Correct / incorrect response	Cloze procedure; multiple choice format; categorize items into correct category	Cloze procedure; multiple choice format; categorize items into correct category	Cloze procedure; multiple choice format; categorize items into correct category	Cloze procedure; multiple choice format; categorize items into correct category	5-point Likert scale	Not reported	Identify the correct response (one item), or 4- or 5-point Likert scale	Binary self-report option: I do know / I do not know	Either a 5-point Likert scale or four multiple-choice options	4-point Likert scale (*very difficult* to *very easy*)

Note. CHLSalt-HK = Chinese Health Literacy Scale for Low Salt Consumption–Hong Kong; CNLI = Critical Nutrition Literacy Instrument; e-NutLit = Electronic-Nutrition Literacy Tool; FL = food literacy; HEI = Health Eating Index; HL = health literacy; ICC = item characteristic curve; IT-FLS = Italian Food Literacy Survey; NL = nutrition literacy; NLit = Nutrition Literacy Assessment Instrument; NLit-BCa = Nutrition Literacy Assessment Instrument for Breast Cancer; NLit-P = Nutrition Literacy Assessment Instrument-Parents; NLit-S = Nutrition Literacy Assessment Instrument-Spanish; NLQ-JP = Nutrition Literacy Questions for Japanese Population; NLS = Nutrition Literacy Scale; NVS = Newest Vital Sign; PSI = Pearson Separation Index; REALM = Rapid Estimate of Adult Literacy in Medicine; SFLQ = Short Food Literacy Questionnaire; S-TOFHLA = Short Test of Functional Health Literacy in Adults.

**Table 2:** Functional, Interactive, and Critical Literacy Domains Captured in Included Measures

**Domains**	**Name of Instrument**												
	**NLS**	**S-NLS**	**NVS**	**NLit**	**NLit-BCa**	**NLit-S**	**NLit-P**	**CNLI**	**e-NutLiT**	**SFLQ**	**NLQ-JP**	**CHLSalt-HK**	**IT-FLS**
Functional literacy (capacity to obtain, understand, and use factual information)													
Understanding of factors that can enhance or inhibit good health (e.g., ability to identify foods that are high in sugar or fat, understand benefits of dietary fiber)	Yes	Yes	Yes	Yes	Yes	Yes	Yes	–	Yes	Yes	Yes	Yes	Yes
Ability to seek information about nutrition, food, and food preparation	-	-	-	-	-	-	-	-	-	Yes	-	Yes	Yes
Knowledge related to accessing, selecting, preparing and eating foods, and planning for meals	-	-	-	Partial; includes items about household food measurement and food shopping skills	Partial; includes items about household food measurement and food shopping skills	Partial; includes items about household food measurement and food shopping skills	Partial; includes items about household food measurement and food shopping skills	-	-	Yes	-	Yes	Yes
Interactive literacy (capacity to act on information to improve health, and to interact and engage in various forms of communication to obtain, provide, and apply relevant health information)													
Interpersonal communication skills	-	-	-	-	-	-	-	-	-	Yes	-	-	-
Ability to translate declarative knowledge into positive dietary choices	-	-	-	Yes	Yes	Yes	Yes	-	Yes	Yes	-	Yes	Yes
Skills, motivation, and confidence to navigate the food system	-	-	-	-	-	-	-	Yes	-	Yes	-	-	-
Skills and behaviors to access, select, prepare and eat foods, and plan for meals	-	-	-	Partial; consumer food shopping skills	Partial; consumer food shopping skills	Partial; consumer food shopping skills	Partial; consumer food shopping skills	-	-	Yes	-	-	Yes
Capacity to apply nutrition information to improve personal health status	-	-	-	-	-	-	-	-	-	Yes	-	-	Yes
Critical literacy (capacity to critically appraise and reflect on health information, available resources, and advice)													
Ability to evaluate the quality of nutrition information from different sources	-	-	-	-	-	-	-	Yes	Yes	Yes	-	-	-
Ability to assess whether food contributes to healthy diet, and to distinguish between healthy and less healthy options	-	-	-	Yes	Yes	Yes	Yes	Yes	Yes	Yes	-	-	Yes
Willingness to take action to improve nutritional health	-	-	-	-	-	-	-	Yes	-	Yes	-	-	-

Note. CHLSalt-HK = Health Literacy Scale for Low Salt Consumption–Hong Kong Population; CNLI = Critical Nutrition Literacy Instrument; e-NutLit, Electronic-Nutrition Literacy Tool; IT-FLS = Italian Food Literacy Survey; NLit = Nutrition Literacy Assessment Instrument; NLit-BCa, Nutrition Literacy Assessment Instrument for Breast Cancer; NLit-P, Nutrition Literacy Assessment Instrument-Parents; NLit-S, Nutrition Literacy Assessment Instrument-Spanish; NLQ-JP, Nutrition Literacy Questions for Japanese Population; NLS = Nutrition Literacy Scale; S-NLS = Spanish Nutrition Literacy Scale; NVS = Newest Vital Sign; SFLQ = Short Food Literacy Questionnaire.
